# Deciphering the *Salmonella* T6SS toolkit: two decades of research decoding a versatile bacterial weapon

**DOI:** 10.1128/jb.00188-25

**Published:** 2025-06-13

**Authors:** Carlos J. Blondel, Fernando A. Amaya, Felipe Reyes-Méndez, Victoria Soriano-Mora, Carla Vargas, Ayleen Parra, María C. Opazo, Viviana Toledo, María F. Barros-Infante, Carlos A. Santiviago, David Pezoa

**Affiliations:** 1 Instituto de Ciencias Biomédicas, Facultad de Medicina y Facultad de Ciencias de la Vida, Universidad Andrés Bello 28087, Santiago, Chile; 2 Laboratorio de Microbiología, Departamento de Bioquímica y Biología Molecular, Facultad de Ciencias Químicas y Farmacéuticas, Universidad de Chile 14655, Santiago, Chile; 3 Núcleo de Investigación en One Health Facultad de Medicina Veterinaria y Agronomía, Universidad de Las Américas 28059, Santiago, Chile; 4 Department of Chemical Engineering, Biotechnology and Materials, Centre for Biotechnology and Bioengineering (CeBiB), Universidad de Chile 14655, Santiago, Chile; 5 Instituto de Ciencias Naturales, Facultad de Medicina Veterinaria y Agronomía, Universidad de Las Américas 28059, Santiago, Chile; 6 Centro de Investigación en Ciencias Biológicas y Químicas, Universidad de Las Américas, Santiago, Chile; 7 Departamento de Ciencias Químicas y Biológicas, Universidad Bernardo O'Higgins 28042, Santiago, Chile; University of Virginia School of Medicine, Charlottesville, Virginia, USA

**Keywords:** *Salmonella*, T6SS, effector, colonization, regulation

## Abstract

The Type VI Secretion System (T6SS) is a critical fitness and virulence factor of many Gram-negative bacteria. Five T6SS gene clusters have been described in *Salmonella*, each one encoded within different pathogenicity islands (i.e., SPI-6, SPI-19, SPI-20, SPI-21, and SPI-22). The events of gain and loss of these T6SS gene clusters have contributed to shape the genome evolution of different *Salmonella* serotypes. In addition, the differential distribution of T6SS and the ever-increasing repertoire of predicted effector proteins is likely to play an important role in the environmental fitness and pathogenic potential of different *Salmonella* serotypes. This review summarizes the current knowledge on the role played by T6SS in *Salmonella* biology, highlighting the major milestones in the field over the past two decades. We discuss the expanding repertoire of T6SS effector proteins identified to date and examine the current understanding of mechanisms controlling T6SS expression in *Salmonella*, focusing on host-derived cues and regulators involved. Finally, we provide a critical analysis of conflicting reports and suggest future directions for the research in the field. A better understanding of these processes could expand our knowledge of *Salmonella* biology, and the mechanisms behind how this versatile secretion system enables *Salmonella* to thrive in competitive microbial environments and contribute to host colonization.

## INTRODUCTION

The Type VI Secretion System (T6SS) is a contractile multiprotein structure that contributes to the environmental fitness and virulence of many Gram-negative bacteria (1–5). Despite this, there are significant gaps in our understanding of how T6SSs contribute to the life cycle of clinically relevant bacteria such as *Salmonella*. The genus *Salmonella* comprises two species, *S. enterica* and *S. bongori*, and over 2,600 different serotypes (6) which vary significantly in their host range and clinical manifestations (7). It is estimated that *Salmonella* infections account for 95.1 million cases of gastroenteritis per year worldwide (8).

**Fig 1:**
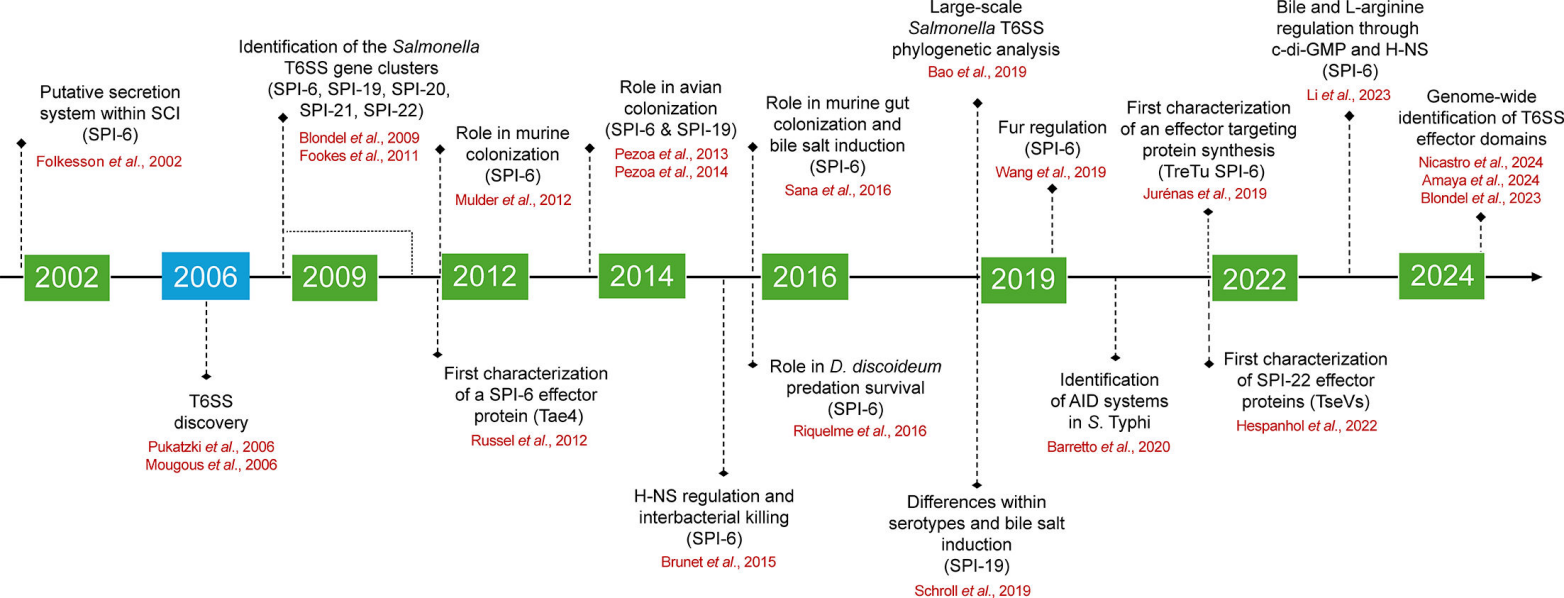
Timeline highlighting key discoveries in T6SS research in *Salmonella*.

Following its initial description over two decades ago (9, 10), studies have underscored the relevance of T6SS to the pathogenic potential and environmental fitness of *Salmonella* (11–26). The recent large-scale identification of novel effector proteins, which exhibit a diverse array of biochemical functions, is further contributing to the expansion of our knowledge regarding the contribution of T6SS to *Salmonella* biology (26–28). This review summarizes the current knowledge regarding the role of T6SS in *Salmonella* and highlights the major milestones in the *Salmonella* T6SS field over the last two decades (Fig. 1), with emphasis on the expanding repertoire of effector proteins, classified according to their predicted targets, and the regulation of T6SS gene expression in response to environmental and host cues.

## THE TYPE VI SECRETION SYSTEM: A VERSATILE NANOMACHINE

The T6SS is a multiprotein nanomachine made of at least 13 structural components and a variable number of accessory proteins that deliver protein effectors into target cells through a contractile mechanism (29–32). The extensive repertoire of effector activities makes the T6SS a highly versatile machine that can target prokaryotic and/or eukaryotic cells (1, 31, 33–47). Briefly, a needle composed of an inner tube (made of Hcp hexamers) tipped by a spike complex comprising VgrG and PAAR proteins is wrapped into a contractile sheath formed by the polymerization of TssB/TssC subunits that is assembled as an extended, metastable conformation (48). Contraction of the sheath upon contact with a target cell or upon sensing cell envelope damage propels the needle toward the target cell (49). Secreted effectors are delivered fused to both VgrG and PAAR proteins (known as evolved or specialized effectors) or through non-covalent interaction with core components such as Hcp (known as cargo effectors) (50–53). Some effectors delivered by T6SS are bacteria-specific, such as those targeting the peptidic or glycosidic bonds of the peptidoglycan (33, 34, 38, 45, 54, 55), or the FtsZ cell division ring (43). Most of these antibacterial effectors are encoded in bicistronic elements with their cognate immunity proteins, thus conforming effector/immunity pairs. Each immunity protein binds tightly and specifically to their cognate effector, preventing self-intoxication and killing of sibling cells (34). Other T6SS effectors are eukaryote-specific, such as those targeting the actin or microtubule cytoskeleton networks (37, 55–68). A third group of T6SS effectors are both bacteria- and eukaryote-specific (trans-kingdom effectors), such as those targeting conserved molecules (e.g., NAD) or macromolecules (e.g., DNA, RNA, and phospholipids), or forming pores in biological membranes (42, 44, 69). Interestingly, it has been reported that enteric pathogens, such as *Salmonella*, *Shigella,* and *Vibrio*, and commensal bacteria, such as *Bacteroides,* employ the T6SS to engage in interbacterial competition in order to colonize the gut (21, 25, 70). Therefore, the T6SS is a key player in bacterial warfare.

## THE T6SS OF *Salmonella*: FROM CRYPTIC PROTEIN SECRETION SYSTEM TO KEY FITNESS AND VIRULENCE FACTOR

**Fig 2:**
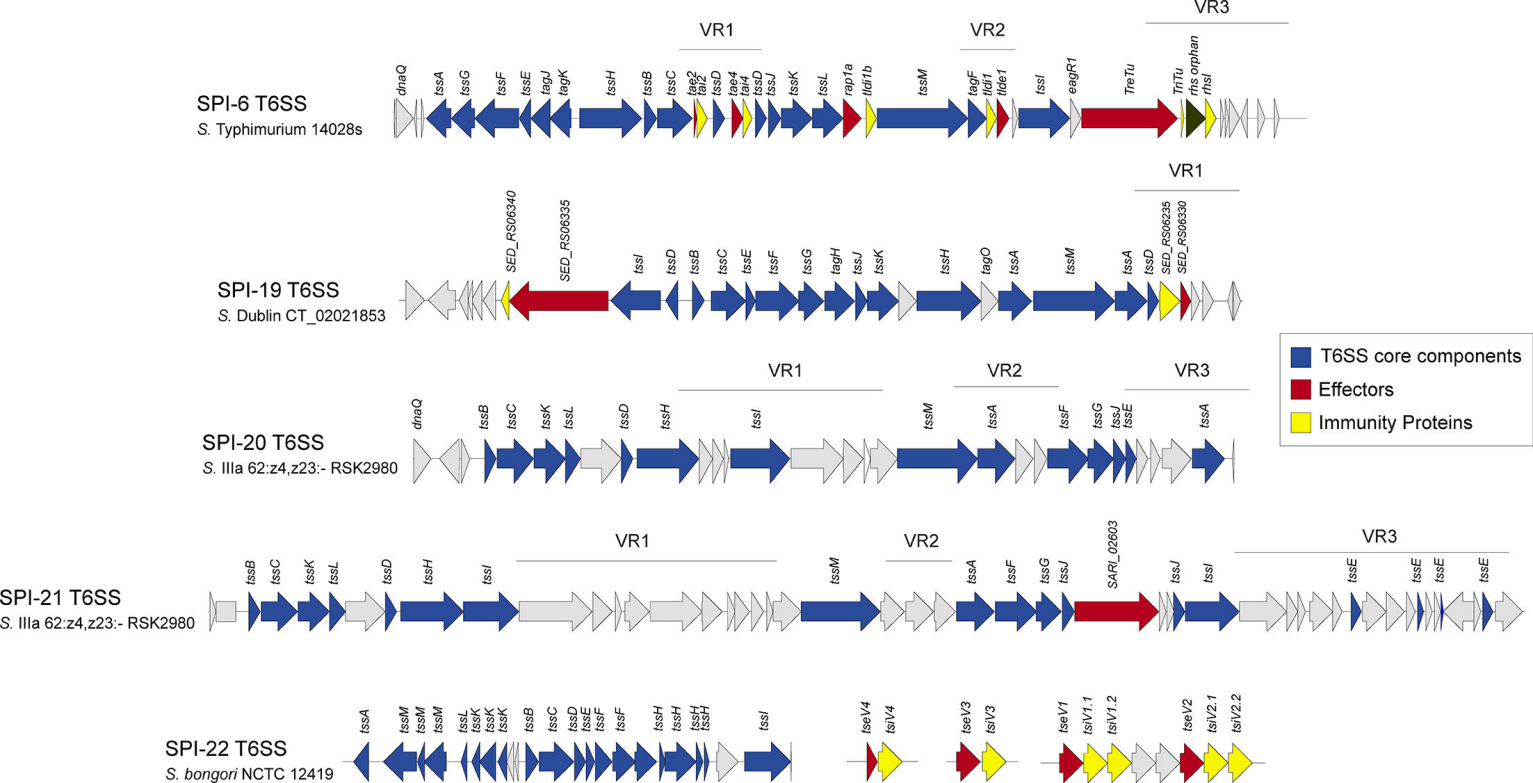
Genetic organization of representative *Salmonella* T6SS gene clusters and their corresponding effector/immunity pairs. Schematic representation of the SPI-6, SPI-19, SPI-20, SPI-21, and SPI-22 T6SS gene clusters in representative *Salmonella* strains. Genes encoding T6SS core components are highlighted in blue, genes encoding effector proteins are highlighted in red, and genes encoding immunity proteins are highlighted in yellow.

The first hint that *Salmonella* encoded a novel secretion system, beyond the well-characterized Type 3 Secretion Systems (T3SS) encoded within pathogenicity islands SPI-1 and SPI-2, came from the initial observation on the presence of a putative secretion system encoded within the “*Salmonella enterica* centisome 7 genomic island” (SCI, currently named as SPI-6) in the vicinity of the tRNA-encoding gene *aspV* (71) (Fig. 1). Soon after the discovery of T6SS in *Vibrio cholerae* and *Pseudomonas aeruginosa* in 2006 (72, 73), comparative genomic analyses revealed that the genus *Salmonella* harbors five T6SSs encoded within pathogenicity islands SPI-6, SPI-19, SPI-20, SPI-21, and SPI-22 (9, 74) (Fig. 2).

Foundational studies by Russel et al. provided the first characterization of a *Salmonella* T6SS effector by heterologous expression in *E. coli* (34). Next, the work by Brunet et al. and Sana et al. demonstrated the antibacterial activity of the T6SS encoded in SPI-6 (T6SS_SPI-6_) in *S*. Typhimurium and shed light into key mechanisms that regulate T6SS expression in *Salmonella* (25, 75), paving the way for further studies exploring the contribution of T6SS to *Salmonella* biology. In the subsequent decade, a large number of studies have established a strong connection between the *Salmonella* T6SSs and key processes such as interbacterial competition and host colonization (11–15, 17–24, 26). In addition, several conflicting reports have linked the T6SSs to *Salmonella* interaction with eukaryotic cells (10–12, 18, 19, 22, 76). The subsequent sections will offer a concise synopsis of these studies.

## PHYLOGENETIC DISTRIBUTION OF T6SS GENE CLUSTERS IN *Salmonella*

The *Salmonella* T6SS gene clusters are distributed in four different evolutionary lineages. SPI-6 T6SS belongs to subtype i3, SPI-19 T6SS to subtype i1, SPI-22 T6SS to subtype i4a, and both SPI-20 T6SS and SPI-21 T6SS belong to subtype i2 (77). Besides their distinct evolutionary origin, these five T6SS gene clusters are differentially distributed among distinct serotypes, subspecies, and species of *Salmonella* (9, 77). SPI-6 T6SS is widely distributed in *Salmonella enterica*, while SPI-19 T6SS is mostly present in strains of serotypes such as *S*. Dublin, *S*. Weltevreden, *S*. Agona, *S*. Gallinarum, and *S*. Pullorum, which generally lack SPI-6 T6SS. On the other hand, SPI-20 T6SS and SPI-21 T6SS are restricted to *S. enterica* subsp. *arizonae* and/or *diarizonae*, while SPI-22 T6SS is restricted to *S. bongori* (9, 74, 77).

The differential distribution and evolutionary lineages of the *Salmonella* T6SS gene clusters suggest that they were acquired through independent horizontal gene transfer events, as has been suggested for other SPIs (78). Furthermore, the acquisition and loss of these gene clusters might have played an important role in the evolution of *Salmonella*. In agreement with this notion, ancestral *S*. Enteritidis strains harbor both the SPI-6 and SPI-19 T6SS gene clusters, whereas those that gave rise to the ancestors of the *S*. Gallinarum complex harbors degenerate versions of SPI-6 T6SS. Furthermore, modern *S*. Enteritidis strains commonly associated with animal and human infection to date harbor degenerate versions of both SPI-6 and SPI-19 T6SS (79). In the case of *S*. Dublin, a recent study reported that a large deletion caused the loss of the SPI-19 T6SS gene cluster in the ST74 lineage during its divergence from the primary ST10 lineage (80). Finally, it has been reported that a strain from a distinct phylogenetic lineage of *S. bongori* acquired a T6SS gene cluster similar to SPI-19 in addition to the SPI-22 T6SS cluster commonly found in representatives of this species (81).

Overall, the implications of the acquisition and loss of T6SS gene clusters on the environmental fitness and pathogenic potential of *Salmonella* are still largely unknown, representing promising avenues for future research.

## CONTRIBUTION OF *Salmonella* T6SSs TO HOST COLONIZATION

It has been shown that T6SS_SPI-6_ of serotypes *S*. Typhimurium, *S*. Typhi, and *S*. Dublin contribute to host colonization in murine and avian models of infection (10–12, 14, 25, 26, 82, 83) (Table 1). Two of these studies established that the role for T6SS_SPI-6_ in *S*. Typhimurium gut colonization is directly linked to T6SS-dependent killing of commensal bacteria (25, 26).

**TABLE 1:** Contribution of T6SS and selected effectors to *Salmonella* biology

T6SS	Serotype	Associated phenotypes	Effector(s) involved	Selected references
SPI-6	*S*. Typhimurium	Intestinal colonization and systemic spread in mice and chickens	Tae4/Rhs^orphan^	(10–13)
		Intracellular replication in avian and murine macrophages	Unknown	(10, 11, 84)
		Intracellular survival in environmental amoeba	Unknown	(16)
		Interbacterial competition	Tae4/Rhs^orphan^/Tlde1/Tge2^*a*^	(17, 24, 25, 75)
		Interbacterial competition and gut colonization in mice	Tox-Act1	(26)
	*S*. Dublin	Intestinal colonization and systemic spread in mice and chickens	Unknown	(14)
		Interbacterial competition	SED_RS01930	(15)
	*S*. Typhi	Infection and systemic dissemination in humanized mice	Unknown	(82)
		Invasion and toxicity in human epithelial cells	Unknown	(22)
SPI-19	*S*. Dublin	Intestinal colonization of mice	Unknown	(18)
		Interbacterial competition	SED_RS06235/SED_RS06335	(15, 18)
	*S*. Gallinarum	Cytotoxicity to primary macrophages from hens	Unknown	(18)
		Gastrointestinal and systemic colonization in chickens	Unknown	(23)
	*S*. Pullorum	Survival within avian macrophages	Unknown	(19)
		Intestinal colonization and systemic spread in chickens	Unknown	(19)
SPI-20	*S. enterica* subsp. *arizonae* serotype 62:z4,z23:-	Unknown	Unknown	(9)
SPI-21	*S. enterica* subsp. *arizonae* serotype 62:z4,z23:-	Predicted interbacterial competition	SARI_02603^*a*^	(9, 85)
SPI-22	*S. bongori*	Interbacterial competition	TseV2/TseV3	(20)

^*a*^ Antibacterial effector proteins encoded within T6SS gene clusters whose T6SS-dependent activity have not been experimentally confirmed.

Regarding T6SS_SPI-19_, some studies have demonstrated its requirement for efficient colonization of mice by *S*. Dublin (18), and chickens by *S*. Gallinarum and *S*. Pullorum (19, 23). In contrast, other authors have not observed a contribution of T6SS_SPI-19_ to chicken colonization by *S*. Gallinarum (18). A possible explanation for this discrepancy could be the use of different bacterial and chicken strains. It is conceivable that differences among strains of the same serotype may exist regarding the contribution of their T6SSs to *Salmonella* pathogenesis as well as differences in the microbiota of different chicken strains. Remarkably, it has been reported that the transfer of the SPI-19 T6SS gene cluster of *S*. Gallinarum into a *S*. Typhimurium mutant strain lacking SPI-6 T6SS complements its colonization defect in both mice and chickens, indicating that T6SS_SPI-6_ and T6SS_SPI-19_ may be functionally redundant during host colonization (13, 86). Finally, despite the established association of T6SS_SPI-6_ and T6SS_SPI-19_ with animal colonization, the repertoire of effector proteins responsible for these phenotypes has not been fully dissected.

## THE MAJOR ROLE OF *Salmonella* T6SSs IN BACTERIAL ANTAGONISM

Up to date, T6SS_SPI-6_, T6SS_SPI-19_, and T6SS_SPI-22_ have been demonstrated to possess antibacterial activity against different Gram-negative species (15, 17, 18, 20, 25). Sana et al. showed that *S*. Typhimurium can use T6SS_SPI-6_ to outcompete representative strains of the gut microbiota such as *Klebsiella oxytoca* and *Klebsiella variicola* but not others such as *Prevotella copri*, *Parabacteroides distasonis*, *Bacteroides fragilis,* and *Bifidobacterium longum* (25). Notably, while *S*. Typhimurium can use T6SS_SPI-6_ to outcompete derivatives of *E. coli* K-12 (DH5α or W3110), it failed to outcompete the *E. coli* JB2 commensal strain, suggesting cross-protection due to uncharacterized immunity proteins (25). Soon after this foundational study, T6SS_SPI-19_ and T6SS_SPI-22_ have also been demonstrated to possess antibacterial activity against different Gram-negative species (15, 17, 18, 20, 25). More recently, it has also been reported that *S*. Typhimurium can directly decrease the sporulation frequency of *Bacillus subtilis* through T6SS-dependent antagonism (87), highlighting different adaptive strategies of *Salmonella* within microbial communities.

T6SS_SPI-19_ has been shown to contribute to interbacterial competition of *Salmonella*, but with remarkable differences among serotypes. While T6SS_SPI-19_ is necessary for *S*. Dublin strains to outcompete derivatives of *E. coli* K-12 (15, 18), this is not observed in the case of *S*. Gallinarum and *S*. Pullorum strains (18, 19, 76). These differences can be attributed to variations in the repertoires of effector/immunity pairs encoded within different SPI-19 T6SS gene clusters, or different experimental conditions used by the authors to induce the expression of genes encoding T6SS_SPI-19_.

T6SS_SPI-22_ has also been shown to contribute to interbacterial competition by *S. bongori* against derivatives of *E. coli* K-12 (20). Although *S. bongori* is commonly associated with cold-blooded animals, it has been reported to infect humans (74). In this context, it remains to be determined whether this system plays a role in colonization of the gut of cold-blooded animals, in human infection, or in the environmental fitness of *S. bongori*. Finally, although there is no experimental evidence on the role played by T6SS_SPI-21_ in interbacterial competition, the presence of an evolved VgrG protein with a C-terminal extension with homology to S-type pyocins encoded in the SPI-21 T6SS gene cluster of *S. enterica* subsp. *arizonae* strain RSK2980 suggest an antibacterial activity as well (9).

## DOES *Salmonella* USE THE T6SS TO TARGET EUKARYOTIC CELLS?

In contrast to what it is known on the contribution of *Salmonella* T6SSs to bacterial antagonism, there are conflicting reports regarding the role played by T6SS in the interaction of *Salmonella* with eukaryotic cells. For example, some reports have shown that deletion of genes *tssM* (*icmF*) and *tssH* (*clpV*), encoding structural components of T6SS_SPI-6_, resulted in a modest decrease in the intracellular replication of *S*. Typhimurium in RAW264.7 murine macrophages (11, 12), and in a slight reduction of cell invasion and cytotoxicity toward HeLa cells (22). In contrast, another study showed that the deletion of *tssM* (*icmF*) resulted in increased intracellular replication of *S*. Typhimurium in J774 murine macrophages (10). Furthermore, genome-wide screens of transposon mutants have consistently failed to identify SPI-6 T6SS-related genes as relevant for *S*. Typhimurium (88, 89) and *S*. Typhi (90, 91) survival within infected macrophages.

Similar discrepancies have been observed regarding T6SS_SPI-19_. Some studies have shown that the deletion of the whole SPI-19 island or genes encoding T6SS structural components such as *tssH* (*clpV*) caused a reduction in invasion and/or intracellular survival of *S*. Gallinarum and *S*. Pullorum within immortalized avian epithelial and macrophage cell lines (19, 76). In contrast, other authors have reported that deletion of SPI-19 does not impair the intracellular survival of *S*. Gallinarum within HD11 chicken macrophages (18).

These discrepancies are not restricted to the interaction of *Salmonella* with avian epithelial cells and macrophages but extend to the possible contribution of T6SS_SPI-6_ in predation survival against environmental eukaryotes, such as amoebae (Table 1). *Dictyostelium discoideum* is a predatory eukaryotic host that has been shown to be targeted by the T6SS of other bacteria, such as *V. cholerae* (58, 72) and *Burkholderia cenocepacia* (92). In this context, a *S*. Typhimurium strain lacking the T6SS_SPI-6_ core component TssH (ClpV) showed a reduced intracellular survival in this social amoeba (16). However, *S*. Typhimurium strains lacking any of the three paralogs of the T6SS_SPI-6_ core component TssD (Hcp) showed no intracellular survival defect in this host (93).

There are several plausible explanations for the discrepancies mentioned above. First, the contribution of the T6SS to the interaction with host cells may be, indeed, modest and obscured by variations in the experimental methodology used by different research groups (e.g., choice of bacterial strains, cell lines, and infection conditions, among others). Alternatively, the phenotypes observed could be the result of a general stress response triggered by the destabilization of the T6SS caused by the deletion of genes encoding structural components, as seen for other protein secretion systems. In support of this notion, a recent study found that a *S*. Typhimurium strain lacking TssH (ClpV) exhibited significantly reduced swimming motility (83). Lastly, these discrepancies could also be linked to differences in the repertoire of T6SS effector proteins carried by strains characterized in different studies.

In addition to these conflicting reports, there is currently no evidence of the delivery of T6SS effector proteins into eukaryotic cells infected by *Salmonella*. These issues represent significant knowledge gaps that require further study.

## THE EXPANDING REPERTOIRE OF *Salmonella* T6SS EFFECTORS

Soon after the discovery of the T6SS gene clusters in *Salmonella*, several studies have aimed to unveil the repertoire of T6SS effector proteins encoded within these clusters in both clinical and environmental strains (26–28). One of these studies corresponds to the largest bioinformatics analysis performed to date to identify T6SS effectors encoded in *Salmonella* genomes included in the 10K genome database (26). This analysis resulted in the identification of more than a hundred candidate effector proteins carrying toxin domains with potential antibacterial activity, considerably expanding our knowledge on the diversity of T6SS effectors (26). Overall, these studies provided a useful framework for the future analysis of larger, more complex, and geographically diverse databases. However, as the number of T6SS effector proteins identified across *Salmonella* genomes continues to grow, there is a pressing need to establish a standardized nomenclature to avoid multiple names being assigned to the same effector, which can result in misleading information and duplication of effort during the experimental validation process. Such endeavors have been proposed for effector proteins of other secretion systems (94).

**Fig 3:**
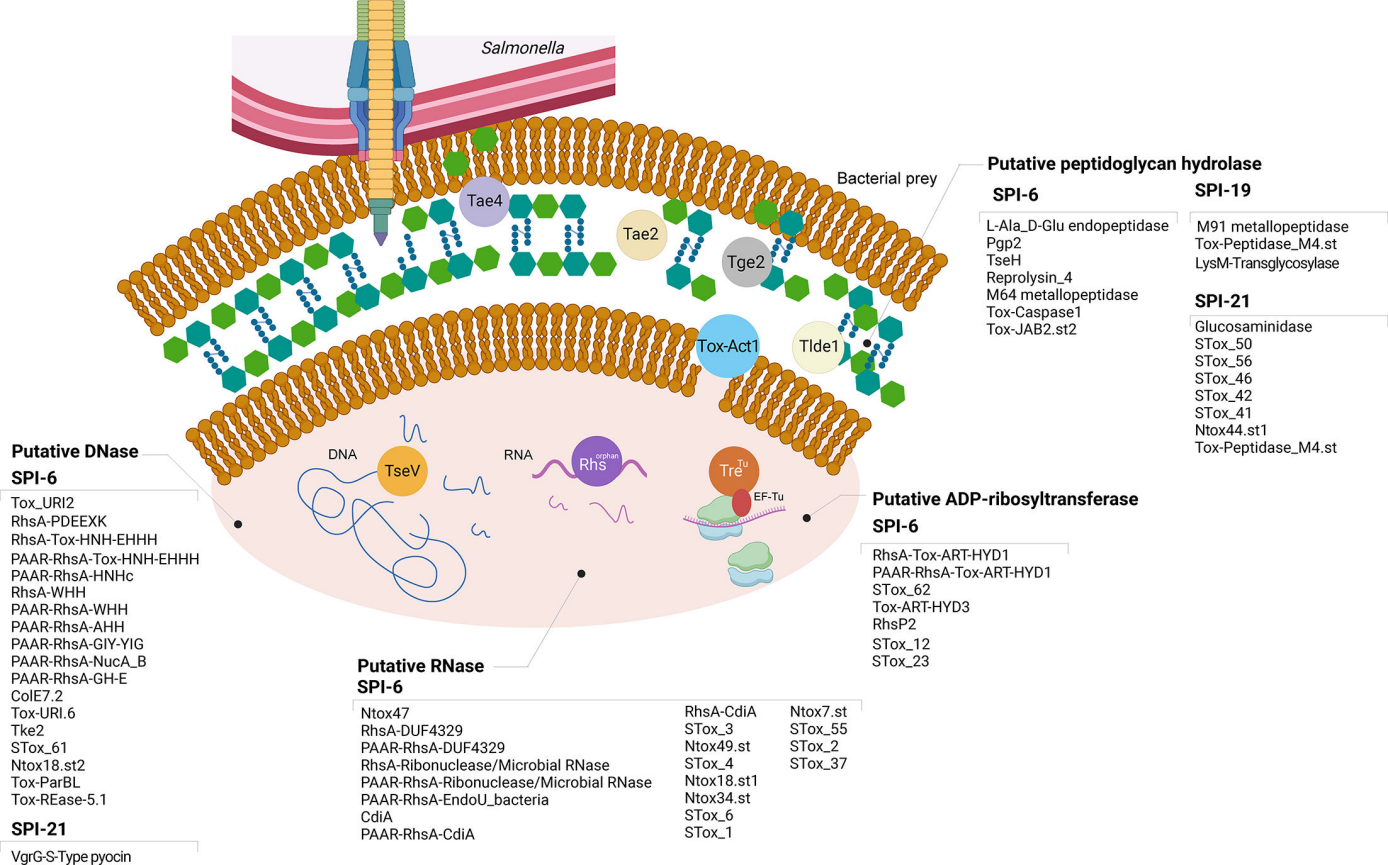
Molecular targets of selected *Salmonella* T6SS antibacterial effectors. Scheme showing the predicted toxic functions of confirmed and candidate effectors.

The diverse predicted targets of the T6SS effectors, which include the cell wall, nucleic acids, biological membranes, and the translation machinery, showcase the complexity and diversity of *Salmonella*’s arsenal (Fig. 3). In the following sections, we provide an overview of different T6SS effectors in terms of their predicted targets.

## EFFECTORS TARGETING THE PEPTIDOGLYCAN LAYER

### SPI-6 encoded effectors

To date, most predicted and characterized T6SS antibacterial effectors in *Salmonella* correspond to cargo effectors encoded within variable regions VR1 and VR2 of the SPI-6 T6SS gene cluster (15, 26, 28). The first biochemically characterized effector in *Salmonella* was Tae4, a D,L-endopeptidase that cleaves the γ-D-glutamyl-L-meso-diaminopimeil acid (mDAP) amid bond of the peptidoglycan (34). Tae4 is part of a family of at least four members (Tae1–Tae4) with different specificities in terms of the peptide cross-bridges they hydrolyze (34). Each of these effectors is encoded with their cognate immunity protein to prevent self-intoxication. After the initial characterization of the Tae family of effectors, two other conserved effectors were soon characterized: the L,D-transpeptidase Tlde1 and the glycoside hydrolase Tge2 (17, 54, 95). These effectors are widely distributed among *Salmonella* and are encoded within SPI-6 VR2.

Recent genome-wide efforts have expanded the repertoire of effectors targeting the peptidoglycan with the identification of numerous candidates encoded within SPI-6 VRs in different *Salmonella* serotypes (26–28), highlighting the importance of the cell wall as one of the main targets for *Salmonella* T6SS effectors.

### SPI-19 encoded effectors

Unlike SPI-6, only three genes encoding effectors targeting the peptidoglycan layer have been identified within SPI-19. One of these effectors includes a Tox-Peptidase_M4 domain present in many metallopeptidases (26), the second one includes LysM and transglycosylase domains frequently found in T6SS antibacterial effectors (34), and the third one carries a N-terminal PAAR domain and a C-terminal M91 metallopeptidase domain (15). Recent experimental evidence indicates that the latter two effectors contribute to the antibacterial activity of T6SS_SPI-19_ in *S*. Dublin. In addition, the antibacterial activity of both effectors is counteracted by their cognate immunity proteins (15).

### SPI-21 encoded effectors

Genes encoding T6SS effectors targeting the cell wall have also been identified in SPI-21, including proteins with predicted glucosaminidase, glicosidase, and metallopeptidase activities (26, 27) even though none of them have been shown to contribute to the antibacterial activity of *S. enterica* subsp. *arizonae* strains.

## EFFECTORS TARGETING NUCLEIC ACIDS

### SPI-6 encoded effectors

Most *Salmonella* effectors predicted to target nucleic acids are encoded within variable region VR3 of the SPI-6 T6SS gene cluster (26–28). Most of these effectors correspond to rearrangement hot spot (Rhs) proteins with C-terminal extensions. These extensions comprise a remarkable variety of endonuclease domains, including DNases, RNases, and deaminases (15, 27, 28, 36). The diversity of these effectors is driven by homologous recombination between *rhs* genes associated with C-terminal displacement, which generates a large repertoire of effector domains (96, 97). This explains how Rhs elements are a significant source of *Salmonella* T6SS effector diversity (96). The first identified T6SS_SPI-6_ effector protein with nuclease activity was the Rhs orphan protein encoded in gene *STM14_0341*, which harbors the Ntox47 RNase domain (36). This effector has been shown to contribute to interbacterial competition and mice colonization in *S*. Typhimurium (36). More recently, a few bioinformatics analyses have identified over a dozen of different DNase, RNAse, and deaminase protein domains in candidate T6SS_SPI-6_ effectors differentially distributed among *Salmonella* genomes (26–28).

### SPI-21 encoded effectors

The first T6SS effector with a putative nuclease domain in *Salmonella* was identified encoded within the SPI-21 T6SS gene clusters of *S. enterica* subsp. *arizonae* and *S. enterica* subsp. *diarizonae* (9). The *SARI_02603* ORF encodes an evolved VgrG protein with a pyocin domain (S-Type) fused at its C-terminus (9). While its role in the antibacterial activity of T6SS_SPI-21_ remains to be tested, Ho et al. replaced the C-terminal domain of VgrG3 in *V. cholerae* V52 with the pyocin domain of the VgrG of *S. enterica* subsp. *arizonae* 62:z4,z23:- (85). The resulting strain expressing the chimeric VgrG3 (i.e., VgrG-NucSe1) killed the mutant strain of *V. cholerae* used as prey in bacterial competition assays, indicating that the S-Type pyocin is a functional T6SS antibacterial domain (85).

### SPI-22 encoded effectors

Recently, a novel family of antibacterial effectors with DNase activity was identified encoded within SPI-22 of *S. bongori* (TseV1, TseV2, and TseV3) (20). These effector proteins harbor an N-terminal PAAR-like domain next to a VRR-Nuc endonuclease domain with structure-specific nuclease activity. Upon delivery, all three effectors cause cell death via double-stranded DNA breaks (20).

## EFFECTORS TARGETING BIOLOGICAL MEMBRANES

To date, a variety of membrane-targeting effectors with predicted lipase and pore-forming activities have been identified encoded within SPI-6 and SPI-21 (26, 27, 35). The predicted lipases include members of the Tle superfamily, harboring toxin domains including the GxSxG and dual HxKxxxxD catalytic motifs (35), and the Tox-Act1 family of phospholipases, harboring a permuted NlpC/P60 domain (26). Tox-Act1 is encoded with its cognate immunity protein (Imm-Act1) in the variable region VR2 of SPI-6 T6SS, and it has been shown to contribute to interbacterial competition in the mouse gut (26).

Interestingly, effectors with predicted pore-forming activity have also been detected encoded within SPI-19 and SPI-21 (26, 27). These effectors include proteins with toxin domains such as NTox_17, STox_18, and Ssp6 (26), and proteins similar to the T6SS antibacterial effectors VasX of *V. cholerae* and BTH_I2691 from *Burkholderia thailandensis*. The predicted structures of VasX and BTH_I2691 resemble that of colicin Ia (34, 35, 98), a bactericidal protein that forms a voltage-dependent channel in the inner membrane of target cells (99).

## EFFECTORS TARGETING BACTERIAL PROTEIN SYNTHESIS

Encoded within SPI-6 is the Rhs protein TreTu (type VI ribosyltranferase effector targeting EF-Tu), which is the founding member of a family of Rhs polymorphic toxins with ADP-ribosyltransferase (ART) activity that inhibit protein synthesis in targeted bacteria (100). In *S*. Typhimurium, TreTu has been reported to ADP-ribosylate EF-Tu, thereby blocking protein synthesis (100). In addition, recent bioinformatics analyses have identified six additional toxin domains encoding putative ARTs with predicted antibacterial activity, including Tox-ART-HYD1.st, Tox-ART-HYD3.st, RhsP2.st, STox_62, STox_23, and STox_12 (26, 28). Further work is required to confirm the toxic activity of these novel effectors and if this activity is linked to the inhibition of protein synthesis.

## PREDICTED ANTIEUKARYOTE EFFECTORS

While there is no experimentally confirmed *Salmonella* T6SS effector with antieukaryotic activity identified to date, several candidate effectors encoded within SPI-6 and SPI-19 have been categorized as putative antieukaryote effectors due to their predicted enzymatic activity and the lack of cognate immunity proteins. In addition, other candidates have been categorized as putative trans-kingdom effectors predicted to target both bacterial and eukaryotic cells (26).

Given the ongoing debate about the role of the T6SS in the interaction of *Salmonella* with eukaryotic cells, the experimental validation and molecular characterization of these predicted effectors is of the utmost importance. This includes analysis of the translocation of these effectors into host cells during infection, the identification of their cellular targets, and the assessment of their overall contribution to the interaction of *Salmonella* with epithelial and immune cells.

## ORPHAN EFFECTORS AND IMMUNITY PROTEINS

Several effector/immunity pairs have been identified encoded outside the main T6SS gene clusters, in the proximity of mobile genetic elements. The first “orphan” T6SS-related proteins described in *Salmonella* were the Hcp-like and Rhs-CT effector proteins (9, 36). More recently, a new family of orphan polymorphic T6SS effectors in Enterobacterales, known as the PIX domain, has also been identified in *Salmonella* (101), contributing to the diversity and flexibility of T6SS function in this pathogen. In addition to effector proteins, there is increasing evidence that bacteria can acquire and integrate T6SS-related immunity genes into their genomes, conferring immunity to T6SS effectors of other strains and species (5, 102). These genes are often referred to as “acquired interbacterial defense” (AID) systems (5). In the case of *Salmonella*, an AID islet (SAIDI) has been identified in *S*. Typhi (103). This SAIDI encodes an IS1 transposase together with eight putative immunity proteins to T6SS effectors and/or contact-dependent growth inhibition (CDI) toxins (103). Thus, the presence of this SAIDI suggests that *S*. Typhi strains may have a fitness advantage over competing bacteria carrying T6SS and/or CDI toxins.

## REGULATION OF T6SS EXPRESSION IN *Salmonella*

The expression of T6SS gene clusters is tightly regulated in other bacterial species, mostly under the influence of different two-component systems, quorum sensing signals and transcriptional regulators, in addition to post-translational regulation mechanisms, among others (104–106). In contrast, the environmental and/or host cues that induce T6SS gene expression in *Salmonella* remained largely unknown for almost a decade after their initial discovery.

**Fig 4:**
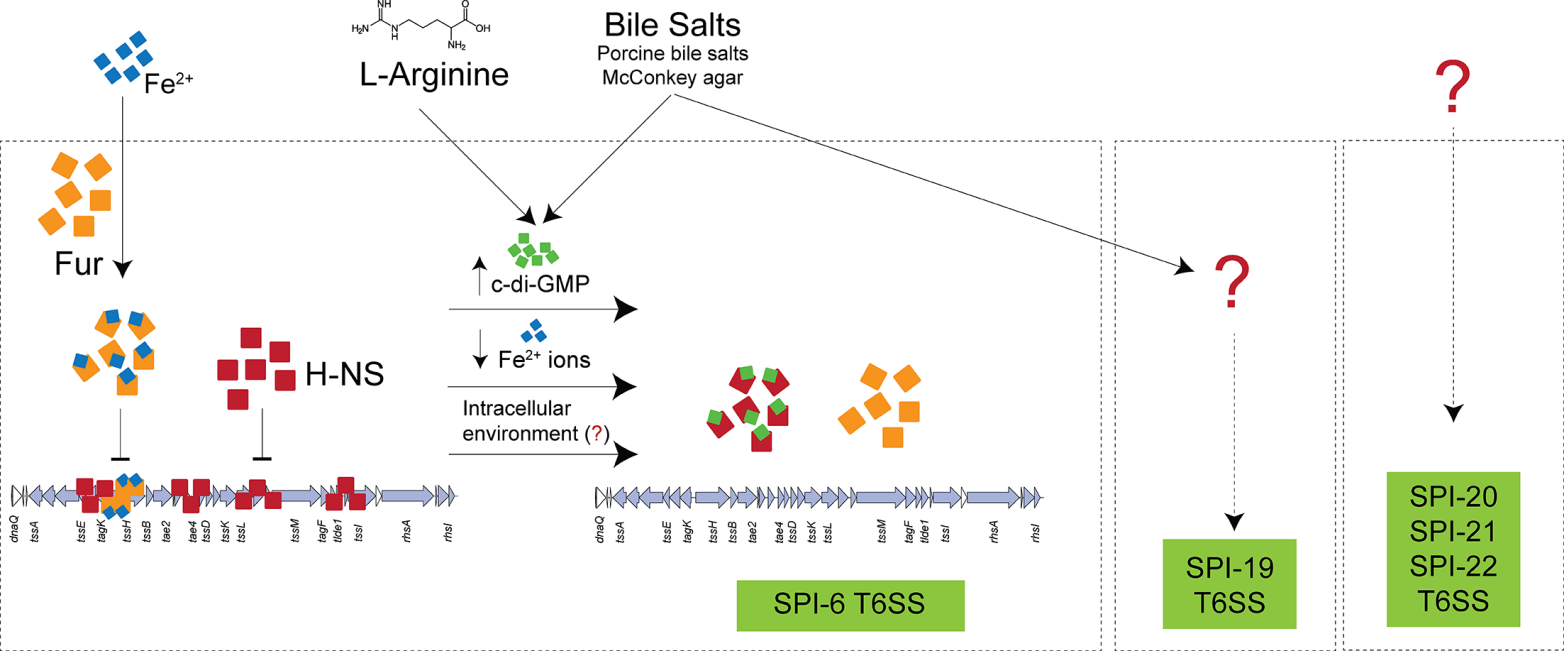
Scheme highlighting the current knowledge on T6SS gene regulation in *Salmonella*. Arrow heads and brackets represent positive and negative regulation, respectively. Solid lines indicate experimentally confirmed direct regulatory effects, and dashed lines indicate indirect regulatory effects via unknown mechanisms. Question marks indicate unknown regulatory signals.

Most of what we currently know regarding T6SS regulation in *Salmonella* comes from studies of the SPI-6 T6SS gene cluster in *S*. Typhimurium and *S*. Typhi (Fig. 4). In the case of *S*. Typhimurium, initial studies revealed that genes within SPI-6 T6SS were upregulated during the infection of macrophages (10, 11, 107). These studies included microarray-based transcriptomic analysis and more focused techniques such as RT-qPCR, analysis of transcriptional reporter fusions, and detection of T6SS proteins (i.e., Hcp and VgrG) by Western blot. In contrast, a subsequent RNA-seq analysis did not show induction of SPI-6 T6SS genes during infection of *S*. Typhimurium in murine macrophages (108). This discrepancy may be attributed to the varying degrees of sensitivity exhibited by the techniques used in the mentioned studies. Regarding *S*. Typhi, the expression of selected SPI-6 T6SS genes was reported to be under the control of the PmrA/PmrB two-component system and the transcriptional regulator RcsB (22). However, the results presented in this study must be interpreted with caution, as the observed differences are modest and require further validation.

From a historical perspective, the first host-derived cue shown to regulate T6SS activity in *Salmonella* was bile salts (Fig. 4). In a key study in *S*. Typhimurium, Sana et al. identified that the antibacterial activity exerted by T6SS_SPI-6_ against members of the intestinal microbiota depended on the presence of porcine bile salts in the culture medium (25). Later, it was shown that bile salts present in MacConkey agar induced the expression of SPI-19 T6SS genes (18) and was required for antibacterial activity in *S*. Dublin (15, 18).

In addition to bile, other host-derived cues such as iron availability and L-arginine have been shown to induce SPI-6 T6SS gene expression in *S*. Typhimurium (Fig. 4) (109, 110). In this case, the regulation in response to iron availability is dependent on the ferric uptake regulator (Fur) (109), and sensing of L-arginine relieves the repression of SPI-6 T6SS genes mediated by the histone-like nucleoid-structuring (H-NS) protein (110). Regarding H-NS, early ChIP-on-chip and transcriptomic experiments indicated that this protein could bind several regions within SPI-6, causing the repression of SPI-6 T6SS genes under standard culture conditions (111, 112). A subsequent study confirmed the H-NS-dependent repression of SPI-6 T6SS genes and that the deletion of *hns* induces T6SS-dependent intoxication of prey bacteria in competition experiments (113). Notably, it was recently shown that H-NS is a c-di-GMP binding protein and that environmental and host-derived cues such as L-arginine and bile salts increase the intracellular levels of c-di-GMP, promoting the transcription of H-NS-repressed SPI-6 T6SS genes in *S*. Typhimurium (Fig. 4) (110).

Overall, while important discoveries have been made in recent years on the mechanisms involved in the regulation of SPI-6 T6SS gene expression, much remains unknown about such mechanisms in the case of the SPI-19, SPI-20, SPI-21, and SPI-22 T6SS gene clusters.

## CONCLUSIONS AND FUTURE DIRECTIONS

Since the initial discovery of five T6SS gene clusters differentially distributed in *Salmonella*, the T6SS has emerged as an important virulence and environmental fitness factor in this pathogen.

Despite its relevance, much remains to be learned about the molecular mechanisms behind the contribution of T6SS to *Salmonella* biology. In fact, most of our current knowledge on the subject comes from the characterization of T6SS_SPI-6_ and T6SS_SPI-19_ in a few serotypes. In addition, only a small proportion of the effector proteins predicted to date have been experimentally validated and biochemically characterized. Likewise, there is limited information on how the expression of all five T6SS gene clusters is regulated by host and environmental cues, and how the bacteria integrate such signals to coordinate T6SS function with other critical secretion systems, such as the T3SS encoded in SPI-1 and SPI-2. This scenario underscores the significant knowledge gap that exists regarding the individual contribution of these T6SSs to the environmental fitness and pathogenic potential of *Salmonella*.

Although the role of the T6SS in bacterial antagonism has been well established in *Salmonella*, its contribution to the interaction of the pathogen with eukaryotic cells is less clear and should be the focus of future research in the field. To this respect, several issues remain to be addressed, such as confirming the translocation of specific effectors into host cells during *Salmonella* infection and the identification of their targets.

Finally, as the number of available *Salmonella* genomes from geographically diverse locations continues to grow, it is plausible to expect an increase in the bioinformatic identification of novel T6SS effectors in the coming years. This highlights the need to work on a standardized nomenclature for *Salmonella* T6SS effector proteins to facilitate their characterization avoiding potential redundancy in the literature.

Future studies that address the knowledge gaps discussed in this review will expand our understanding of how the T6SS contributes to the environmental fitness and pathogenic potential of *Salmonella* and possibly facilitate the development of more effective intervention strategies to prevent and control infection.
